# Cancer screening information-seeking before and during the COVID-19 pandemic

**DOI:** 10.1080/28322134.2024.2369168

**Published:** 2024-07-01

**Authors:** Heather Platter, Adaora Ezeani, Travis Hyams, Grace C. Huang, William M.P. Klein, Robin C. Vanderpool

**Affiliations:** a Cancer Prevention Fellowship Program, Division of Cancer Prevention, National Cancer Institute, National Institutes of Health, Rockville, MD, USA;; b Office of the Associate Director, Behavioral Research Program, Division of Cancer Control and Population Sciences, National Cancer Institute, National Institutes of Health, Rockville, MD, USA;; c Health Behaviors Research Branch, Behavioral Research Program, Division of Cancer Control and Population Sciences, National Cancer Institute, National Institutes of Health, Rockville, MD, USA;; d Office of the Director, Division of Cancer Control and Population Sciences, National Cancer Institute, National Institutes of Health, Rockville, MD, USA;; e Public Health and Epidemiology, Westat, Rockville, MD, USA;; f Health Communication and Informatics Research Branch, Behavioral Research Program, Division of Cancer Control and Population Sciences, National Cancer Institute, National Institutes of Health, Rockville, MD, USA

**Keywords:** Health information-seeking, cancer screening, COVID-19, pandemic

## Abstract

**Background:**

The COVID-19 pandemic disrupted routine cancer screening; however, it is unclear whether the public’s cancer screening information-seeking behaviors changed. Our study examined cancer screening information-seeking before and after pandemic onset using data from the National Cancer Institute’s Cancer Information Service.

**Methods:**

We analyzed screening inquiries from general public users before (3/27/19–3/26/20) and after (3/27/20–3/26/21) pandemic onset. We examined point of access, subjects of interaction, and referrals.

**Results:**

There were more general public cancer screening inquiries made post-pandemic onset (*n* = 1069; 56.1%) compared to before (*n* = 837, 43.9%). Although the proportion of breast cancer screening inquiries increased after pandemic onset, inquiries for cervical, colorectal, and other cancers decreased. Telephone inquiries increased, whereas email and instant chat inquiries decreased. Inquiries regarding finding healthcare services and managing costs increased, whereas screening tests and other subjects decreased. Referrals increased for the National Breast and Cervical Cancer Early Detection Program and other national/community organizations. All differences were significant at *p* ≤ .05.

**Conclusions:**

We found potentially important changes in cancer screening information-seeking after pandemic onset, including increasing interest in breast cancer screening, yet decreasing interest in other cancer screening tests. Future work should evaluate how public health crises affect information seeking and influence intentions to screen for cancer.

## Introduction

Routine guideline-based screening allows for the early identification of precancerous lesions or established cancers, which improves individual longevity and population health [1]. The COVID-19 pandemic interrupted routine medical care procedures such as cancer screening due to stay-at-home orders, reduced medical facility staffing, cancelation of elective medical procedures, and hesitancy to enter public spaces [2]. Data show that by June 2020, 40% of United States (U.S.) adults reported avoiding medical care, such as cancer screening, due to COVID-19 concerns [3]. Statistical models suggest an excess of 10,000 deaths from breast and colorectal cancer over the next decade due to pandemic screening delays [4].

Cancer information-seeking is an important behavior across the cancer control continuum [5] and seeking information about screening and early detection is associated with improved adherence and timeliness of screening [6]. However, little research has focused on cancer information-seeking behaviors during the COVID-19 pandemic. Vanderpool et al. [7] found that people who inquired about COVID-19 to the U.S. National Cancer Institute’s (NCI) Cancer Information Service (CIS) were significantly more likely to ask about cancer diagnosis, treatment, and post-treatment compared to those who did not inquire about COVID-19. Likewise, while cancer screening information-seeking could be more substantial during the pandemic given potential concerns about missing a screening, information-seeking may decrease due to the shift in health priorities during a heightened state of emergency. It is important to understand information-seeking in the context of health emergencies to adequately prepare for future emergencies.

In this study, we specifically examined cancer screening information-seeking before and after the COVID-19 pandemic onset using data over a 24-month period from the U.S. NCI’s CIS, a well-known and trusted multichannel source of cancer information. We examined CIS point of access (e.g. telephone, instant chat), which describes how the general public contacted the CIS, to determine any differences before and after pandemic onset. We also examined additional topics mentioned by the general public when inquiring about cancer screening, and how CIS referrals to other resources differed during the study period. We hypothesized that cancer screening information-seeking would decrease after the pandemic onset due to interrupted routine medical care procedures and a focus on the pandemic. Findings can be used to contextualize longitudinal, downstream changes in cancer screening, incidence, and mortality following public crises, and have implications for local and state public health agencies to optimize communication of cancer information during future public health emergencies.

## Methods

### Data source

Established by NCI in 1975, the CIS is a publicly available source of accurate, up-to-date cancer information accessible to the general public, health professionals, caregivers, and patients in English and Spanish language [8]. The program provides cancer information through various modalities, such as email (NCIinfo@nih.gov), an instant chat service (LiveHelp), social media (i.e. Face-book, Instagram, LinkedIn, Twitter, YouTube), and telephone (1-800-4-CANCER). Users have access to publications, recorded cancer information, or an information specialist who can answer cancer-related questions and provide individually tailored information [9].

### Study sample

Recorded via an electronic contact record form, CIS information specialists (IS) are trained to code each inquiry using multiple variables, such as the phase of the cancer continuum an inquiry centers on (e.g. screening, diagnosis, treatment) and user type (e.g. cancer survivor, caregiver, general public, health professionals, tobacco user). For the purposes of this analysis, we examined cancer screening inquiries made by general public CIS users as they totaled the majority of all cancer screening inquiries (*n* = 1906, 76.9%). General public CIS users included members of the public or their family members and friends who may have had precancerous or non-cancerous conditions or cancer-related symptoms. The timeline for this study was determined by the first general public CIS user inquiry received about cancer screening after pandemic onset, which occurred on March 27, 2020. Cancer screening inquiries were compared across two equal periods: (1) one year before the COVID-19 pandemic: March 27, 2019-March 26, 2020, and (2) one year after the pandemic onset: March 27, 2020-March 26, 2021. As CIS programmatic data are fully deidentified, related analyses did not qualify as human subjects research by Westat (No. 00005551) and U.S. National Institutes of Health (No. 000815) Institutional Review Boards.

### Measures

We examined several variables to understand information-seeking about cancer screening among general public CIS users before and after pandemic onset (March 27, 2019-March 26, 2020 vs. March 27, 2020-March 26, 2021). Variables included: (1) CIS point of access (email, Live-Help, social media, telephone); (2) subjects of interaction to understand what subjects CIS users inquired about in relation to cancer screening; and (3) types of referrals offered to CIS users, such as where someone can go to get screened. Subjects were aggregated into five categories: (1) ‘finding healthcare services to manage cancer care’; (2) ‘general cancer questions’; (3) ‘managing costs and medical information’; (4) ‘other’; and (5) ‘tests to screen, diagnose, stage, or monitor cancer’. For example, finding healthcare services included finding support, transportation, and lodging for screening services. There were eight users without a subject of interaction code and were treated as missing. IS referrals included: (1) ‘Centers for Disease Control and Prevention National Breast and Cervical Cancer Early Detection Program (CDC NBCCEDP)’; (2) ‘health professional’; (3) ‘national or community organization (includes referrals to community organizations and government programs)’; (4) ‘other (includes any other recommendation that does not fall in the before listed categories)’; and (5) ‘no referral’.

We calculated cancer screening inquiries by general public CIS users before and after the pandemic onset for each cancer site. Cancer sites of interest were categorized as ‘breast’, ‘cervical’, ‘colorectal’, and ‘lung and bronchus’, representing cancer types with A or B screening test recommendations from the U.S. Preventive Services Task Force (USPSTF) [1]. We combined the 28 remaining cancer sites into a single category, ‘other’, to account for cancer screening inquiries without A or B USPSTF ratings. The largest ‘other’ inquiry was prostate cancer (*n* = 84) and 16 ‘other’ cancers had five inquiries or less (*n* = 42). Users who inquired about cancer screening, but did not specify a cancer site, were categorized as ‘general’. For each CIS inquiry, IS could code up to four subjects of interaction categories, three referrals, and two cancer sites.

### Data analysis

We calculated descriptive statistics of cancer screening inquiries from general public CIS users before and after the pandemic onset. To illustrate trends over time, we graphed frequencies. We computed descriptive statistics for point of access, subjects of interaction, and referrals for the pre – and during-pandemic periods. To examine trends over time, we performed bivariate analyses using chi-squared tests of independence to examine the association between these variables and period for each cancer site. We considered associations statistically significant at *p* ≤ 0.05. We conducted analyses with IBM SPSS Statistics Subscription for Mac OS Build 1.0.0.1058.

## Results

Over a 24-month period (March 2019–March 2021), there were 8868 total inquiries to the CIS across the cancer care continuum from general public CIS users. Of these general public inquiries, 1906 inquired about cancer screening: 837 (43.9%) inquiries were pre-pandemic and 1069 (56.1%) during the pandemic.

### Cancer site

Cancer site inquiries across the two periods included breast (*n* = 958), cervical (*n* = 179), colorectal (*n* = 183), general (*n* = 327), lung (*n* = 51), and 28 other cancers combined (*n* = 213). Before the pandemic, breast cancer (*n* = 319, 38.1%), general cancer (*n* = 150, 17.9%), and cervical cancer (*n* 133, 15.9%) accounted for more than two-thirds of screening inquiries. After pandemic onset, breast cancer (*n* = 639, 59.8%) alone made up almost two-thirds of the inquiries, followed by general cancer screening (*n* = 177, 16.6%). The proportion of breast cancer inquiries increased by 21.7% after the pandemic onset (*x*^2^ = 88.13, *p* < .001), the only increase among all cancer types (Table 1).

**Figure 1.**
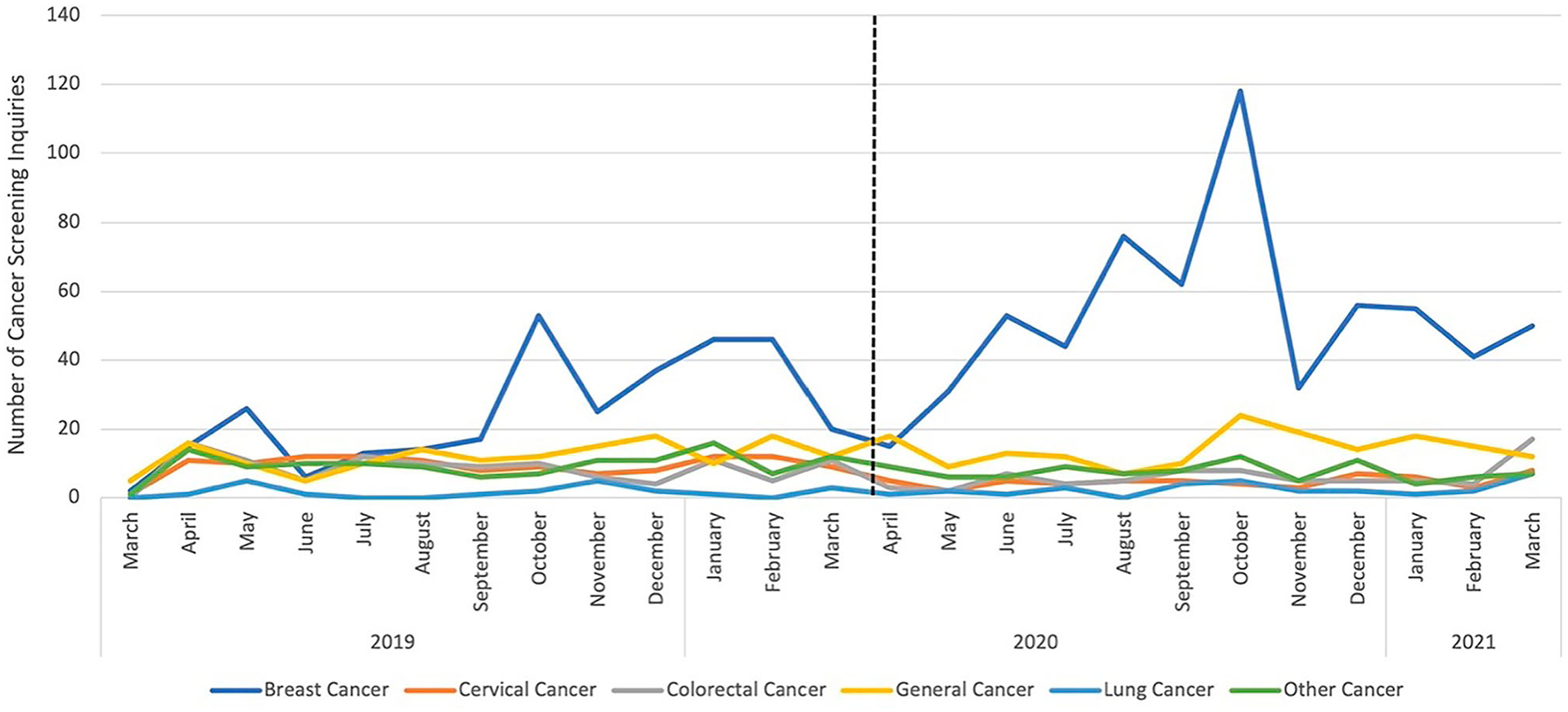
Monthly trend of cancer screening inquiries among general public CIS users pre-pandemic and post-pandemic onset, March 2019–March 2021.

Figure 1 shows the monthly totals of cancer screening CIS inquiries pre – and post-pandemic onset separated by cancer type. Breast cancer screening inquiries peaked in October 2019 (*n* = 53), more than tripling inquiries from September 2019 (*n* = 17). Breast cancer inquiries decreased by 28 in November 2019, then rebounded to 46 across January and February 2020. The decrease may be due to end-of-year holidays in November and December. After the pandemic onset, breast cancer inquiries in June 2020 were more than eight times that of June 2019 and matched October 2019 (Breast Cancer Awareness Month). Inquiries peaked again in October 2020 (*n* = 118), followed by a decline in November 2020 (*n* = 32), which is similar to end-of-year trends from 2019.

### Point of access

General public cancer screening inquiries from telephone calls significantly increased by 14.9% during the pandemic (64.3%; *n* = 538 to 79.2%; *n* = 847; *p* < .001). Inquiries by email decreased by 3.8% (7.4%; *n* = 62 to 3.6%; *n* = 38; *p* < = .001) and LiveHelp decreased by 10.6% (25.1%; *n* = 210 to 14.5%; *n* = 155; *p* < .001). Among cancer sites, breast cancer received the highest proportion of telephone-based inquiries, which increased by 21.3% after pandemic onset (48.1%; *n* = 256 to 69.4%; *n* = 587; *p* < 0.001). The highest proportion of LiveHelp inquiries pre-pandemic was for cervical cancer (31.9%; *n* = 67), but general cancer was higher after the pandemic onset (28.4%; *n* = 44). General cancer had the highest proportion of inquiries for email (27.4%; *n* = 17 vs 36.8%; *n* = 14) and social media (40.7%; *n* = 11 vs 65.5%; *n* = 19) across both periods.

### Subjects of interaction

Three subjects of interaction had notable differences in cancer screening inquiries before and after the pandemic onset: (1) finding healthcare services (*n* = 916); (2) tests (*n* = 530); and (3) managing costs (*n* = 503). There was a significant increase after the pandemic onset of 17.1% (*n* = 272) and 22.6% (*n* = 223) for inquiries coded as finding healthcare services and managing costs, respectively (*p* < 0.001). However, inquiries about tests and other subjects of interactions decreased (*p* < 0.001; Table 1).

When examining subjects of interaction by cancer site, there was a large increase after the pandemic onset for finding healthcare services among breast cancer inquiries (53.1%; *n* = 171 to 77.6%; *n* = 461; *p* < .001). The greatest decrease for finding healthcare services was for colorectal cancer inquiries (18.6%; *n* = 60 to 5.4%; *n* = 32; *p* < .001). The largest increase in general cancer questions was for breast cancer (14.3%; *n* = 18 to 28.7%; *n* = 54; *p* < .003) and the biggest decrease was cervical cancer (26.2%; *n* = 33 to 6.4%; *n* = 12; *p* < .001). For managing costs, the only increase was for breast cancer (52.2%; *n* = 60 to 83.8%; *n* = 325; *p* < .001), whereas the largest decrease was colorectal cancer (26.1%; *n* = 30 to 7%; *n* = 27; *p* < .001). For tests, the biggest increase was for breast cancer inquiries (34.8%; *n* = 96 to 54.3%; *n* = 138; *p* < .001) and the biggest decrease was for other cancers (19.2%; *n* = 53 to 9.8%; *n* = 25; *p* = .002). The only significant change for other subjects of interaction was for cervical cancer (18.7%; *n* = 46 to 10.7%; *n* = 19; *p* = .025) (Table 2).

### Referrals given by CIS staff

The most common IS referral was for a health professional (64.4%; *n* = 539), but this percentage decreased to 52.1% (*n* = 557) after pandemic onset (*p* < 0.001; Table 1). The second highest referral was for national or community organizations (37.8%; *n* = 316), which increased to 60.1% (*n* = 643; *p* < 0.001). Furthermore, 32.8% (*n* = 351) of referrals were to CDC NBCCEDP after pandemic onset, an increase from the 9.3% (*n* = 78) pre-pandemic (*p* < .001). Specifically, CDC NBCCEDP referrals increased for breast cancer (83.3%; *n* = 65 to 93.4%; *n* = 328; *p* < .001) and decreased for colorectal (6.4%; *n* = 5 to 1.4%; *n* = 5; *p* = .008). Referrals to health professionals increased for breast cancer (31.4%; *n* = 169 to 49.2%; 274; *p* < .001) yet decreased for cervical (17.6%; *n* = 95 to 8.8%; *n* = 49; *p* < .001) and colorectal cancer (16.5%; *n* = 89 to 9.2%; *n* = 51; *p* < .001). The only increase for national or community organizations after pandemic onset was for breast cancer (55.7%; *n* = 176 to 79.9%; *n* = 514; *p* < .001), whereas there were decreases for cervical (11.1%; *n* = 35 to 5.3%; *n* = 34; *p* = .001), colorectal (17.4%; *n* = 55 to 6.4%; *n* = 41; *p* < .001), and other cancers (8.2%; *n* = 26 to 4.4%; *n* = 28; *p* = .014). Referrals to other places increased for breast cancer (42.6%; *n* = 124 to 54.3%; *n* = 170; *p* = .004) and decreased for cervical cancer (12.7%; *n* = 37 to 6.4%; *n* = 20; *p* = .008). No referrals showed an increase for general cancer (26.4%; *n* = 23 to 48.1%; *n* = 38; *p* = .004) and a decrease for cervical cancer (20.7%; *n* = 18 to 7.6%; *n* = 6; *p* = .017). It is noteworthy that there was a significant increase in all referral types for breast cancer-related inquiries after the onset of the pandemic (Table 3).

## Discussion

Information-seeking has been shown to be associated with cancer screening adherence [6]; therefore, it may be used as a potential indicator of cancer screening behavior in future public health emergencies. CIS inquiries may demonstrate patterns in cancer screening information-seeking when essential healthcare services are disrupted. We hypothesized that health information-seeking about cancer screening would decrease after the pandemic onset. On the contrary, we found that the proportion of cancer screening inquiries increased by 12.2% (in absolute terms) after pandemic onset. It is notable that this increase was driven primarily by breast cancer inquiries, despite a substantial decrease in the first two months of COVID-19 (March-April 2020). This suggests the majority of inquiries declined at the early onset of the pandemic when hospitals, healthcare centers, and medical providers postponed or canceled non-emergent care and cancer screenings [10]. As with breast cancer, cervical and general cancer inquiries declined in March, whereas colorectal, lung, and other cancer inquiries started to decline in April. Similar studies have used Google Trends to examine public interest in cancer screenings following COVID-19, finding a sharp decline in information-seeking regarding screening tests, such as colonoscopies, human papillomavirus testing, and Pap smears [11,12]. This trend was also similar to previously reported sharp declines and rebound, at least for breast cancer, in cancer screening [13].

We observed a twofold increase in colorectal cancer screening inquiries in March 2020 compared to February 2020, with March 2021 inquiries being more than four times that of February 2021, which coincides with National Colorectal Cancer Awareness Month. We witnessed a peak in breast cancer inquiries during Breast Cancer Awareness Month (October 2019, October 2020), yet October 2020 inquiries were more than twice that of 2019. As stay-at-home emergency orders started to ease by June 2020 [14], breast cancer inquiries matched those in October 2019 (*n* = 53). Hospitals and medical facilities began to reopen, which may have motivated the general public to contact the CIS about cancer screening.

The CIS experienced a significant increase in telephone inquiries after pandemic onset, whereas all other points of access decreased. The surge in telephone inquiries may be due to the increased urgency for expert information and personalized advice on cancer screening during a crisis. Vanderpool et al. [7] found higher telephone usage among general public CIS users, cancer survivors, and tobacco users who inquired about COVID-19 compared to users who did not inquire about COVID-19, indicating that there may be a preference to speak with a ‘live person’ during health emergencies.

Notably, in May 2019, the CIS conducted a communication campaign in the National Library of Medicine Patient Magazine, resulting in an increase of 14,000 inquiries, a majority through LiveHelp. This campaign may account for the higher number of LiveHelp inquiries in 2019.

Study results emphasize the need for local, state, and national public health agencies to strengthen bidirectional information services to communicate essential health information during a crisis [15]. The President’s Cancer Panel on cancer screening recommends using the four-part health literacy framework to develop and disseminate effective communications about cancer screening to empower the public to make decisions about their health [16]. It is important for public health agencies to consider using this framework to develop targeted messaging for individual cancer types and tailor messages to specific populations, especially those with low rates of cancer screening. Furthermore, public health agencies may consider improving the promotion and use of multiple communication channels (e.g. telephone, email) to improve interpersonal communication with the public [7,16]. Interpersonal communication resources via trusted public health organizations and governmental agencies can support the public with information overload, navigating uncertainty, and combating misinformation; provide an outlet to discuss emotional and health-related stressors; and provide referrals to medical, public health, or social service organizations [17,18].

Data demonstrate a significant change in various types of referrals offered to general public CIS users across the study period. The increase in breast cancer inquiries and decrease across other cancer sites after pandemic onset may correspond with the increase in the proportion of referrals to the CDC NBCCEDP. Referrals to programs that serve the uninsured or underinsured, like the CDC NBCCEDP, reduce breast and cervical cancer disparities by providing critical cancer screening and diagnostic services to people with low income and inadequate health insurance [19]. DeGroff et al. [10] examined the impact of the pandemic on the CDC NBCCEDP’s screening services, finding that screening tests declined by 87% in April 2020 for breast cancer. Testing began to recover in May 2020, similar to breast cancer inquiries in this study, although by June it continued to be below the 5-year monthly average [10]. We also found an increase in referrals to national and community organizations or other government programs and a decrease to healthcare providers. Since the beginning of the pandemic, healthcare systems and providers have postponed or canceled services such as elective surgical procedures and cancer screening tests, which may have accounted for the decrease in these referrals [20,21].

### Limitations

Data from this study may not be generalizable to the broader general public as the study was based on a sample of users who proactively contacted a national cancer information service for information about cancer screening. Moreover, given that personal identifiable information is not collected by CIS staff, each contact was treated as a unique user; therefore, it is unknown if any of the users were repeat contacts. Third, sociodemographic data are randomly collected after CIS interactions to minimize respondent burden per White House Office of Management and Budget mandates; these data were excluded from analysis due to small sample sizes within sociodemographic groups. Finally, although the data reported here are quantitative, user inquiries are more qualitative in nature and are coded by trained CIS staff who may be subject to internal biases. To reduce the potential for such biases, they receive extensive training on topics such as patient-centered cancer information in order to code inquiries accurately.

## Conclusion

Cancer screening information-seeking among general public CIS users experienced a sharp decrease following the pandemic onset in March 2020. Breast cancer inquiries quickly rebounded by June 2020, demonstrating an elevated demand for information about breast cancer screening. Furthermore, the increase in breast cancer screening inquiries associated with finding healthcare services reflected a critical period after the pandemic when cancer screening programs were delayed. The observed increase may point to future increases in screening behaviors, requiring healthcare systems to monitor resultant backlog and delay of catch-up screening appointments. Therefore, it is imperative to develop a strategy to optimize cancer screening activities during the pandemic recovery period. In one study, microsimulation models showed – between 2020 and 2060 – continued screening after the maximum recommended age (e.g. after 74 years for breast cancer) had similar cancer incidence and cancer-specific mortality rates as if screening was undisrupted [22]. Furthermore, these data may give insight into the nature of cancer screening information-seeking during future pandemics and public health emergencies, which can help local, state, and federal agencies optimize their communication of cancer information. Future preparation can support the expansion or development of contact access points as well as training staff to clearly communicate health information in times of stress. Special attention should also be paid to guideline-recommended cervical, colorectal, and lung cancer screening tests considering the reduced information-seeking CIS inquiries among the general public. In addition, study results highlight the necessity of enlisting national and community organizations to alleviate the stress of the pandemic on healthcare systems. Connecting CIS users to community and national programs that provide screening and diagnostic services may be an effective method to alleviate barriers in providing cancer screening information and care during national health crises.

## Figures and Tables

**Table 1.** Cancer screening type, point of access, subject of interaction, and referrals by time among general public Cancer Information Service (CIS) users (%), pre-pandemic March 2019 – pandemic March 2021^a^

General public (%)					
	Pre-Pandemic (March 27, 2019 – March 26, 2020)	Pandemic (March 27,2020 – March 26, 2021)	Total	*x*^2^-statistic	p-value
**N**	837	1069	1906		
**Cancer Site of Interest**					
Breast	319 (38.1%)	639 (59.8%)	958	88.13	***p* < .001**
Cervical	133 (15.9%)	79 (7.4%)	212	34.31	***p* < .001**
Colorectal	109 (14.1%)	74 (7.5%)	183	22.06	***p* < .001**
General	150 (17.9%)	177 (16.6%)	327	0.61	*p* = .433
Lung and bronchus	24 (2.9%)	32 (3.0%)	56	0.03	*p* = .872
Other	120 (15.4%)	93 (9.8%)	213	13.62	***p* < .001**
**CIS Point of Access**					
Email	62 (7.4%)	3 8 (3.6%)	100	14.02	***p* < .001**
LiveHelp	210 (25.1%)	155 (14.5%)	365	34.00	***p* < .001**
Social media	27 (3.2%)	29 (2.7%)	56	0.43	*p* = .510
Telephone	538 (64.3%)	847 (79.2%)	1385	52.86	***p* < .001**
**Subjects of Interaction**					
Find healthcare services	322 (38.5%)	594 (55.6%)	916	54.96	***p* < .001**
General cancer questions	126 (15.1%)	188 (17.6%)	314	2.19	*p* = .139
Managing costs	115 (13.7%)	388 (36.3%)	503	122.95	***p* < .001**
Other	246 (29.4%)	177 (16.6%)	423	44.77	***p* < .001**
Tests	276 (33.0%)	254 (23.8%)	530	19.86	***p* < .001**
Missing	2 (0.2%)	6 (0.6%)	8	1.17	*p* = .280
**Referrals**					
CDC NBCCEDP	78 (9.3%)	351 (32.8%)	429	148.83	***p* < .001**
Health professional	539 (64.4%)	557 (52.1%)	1096	29.03	***p* < .001**
Organization	316 (37.8%)	643 (60.1%)	959	94.19	***p* < .001**
Other	291 (34.8%)	313 (29.3%)	604	6.53	***p* = .011**
No referral	87 (10.4%)	79 (7.4%)	166	5.33	***p* = .021**

a CIS users could inquire about multiple cancer sites (up to two), subjects of interaction (up to four), and referrals (up to three); therefore, percentages in some columns may not sum to 100%. Chi-square tests were performed to determine *P* values.

**Table 2.** Subjects of interaction relevant to cancer screening by time and cancer site among general public Cancer Information Service (CIS) users (%), March 2019 – March 2021^a^

	Breast (%)		Cervical (%)		Colorectal (%)		General (%)		Lung (%)		Other (%)	
	Pre-Pan	Pan	Pre-Pan	Pan	Pre-Pan	Pan	Pre-Pan	Pan	Pre-Pan	Pan	Pre-Pan	Pan
N	319	639	113	79	118	80	150	177	24	32	129	105
Find health care services	171 (53.1%)	461 (77.6%)	26 (8.1%)	34 (5.7%)	60 (18.6%)	32 (5.4%)	48 (14.9%)	61 (10.3%)	7 (2.2%)	9 (1.5%)	29 (9.0%)	29 (4.9%)
	**χ**^**2**^ **= 58.61, *p* < .001**		χ^2^ = 1.89, *p* = .17		**χ**^**2**^ **= 40.55, *p* < .001**		**χ**^**2**^ **= 4.28, *p* = .038**		χ^2^ **=** 0.53, *p* = .467		**χ**^**2**^ **= 5.99, *p* = .014**	
General cancer questions	18 (14.3%)	54 (28.7%)	33 (26.2%)	12 (6.4%)	11 (8.7%)	9 (4.8%)	38 (30.2%)	78 (41.5%)	4 (3.2%)	7 (3.7%)	27 (21.4%)	30 (16.0%)
	**χ**^**2**^ **= 8.9, *p* = .003**		**χ**^**2**^ **= 24.11, *p* < .001**		χ^2^ = 1.97, *p* = .161		**χ**^**2**^ **= 4.16, *p* = .041**		χ^2^ = 0.07, *p* **=** .795		χ^2^ = 1.52, *p* = .218	
Managing costs	60 (52.2%)	325 (83.8%)	8 (7.0%)	26 (6.7%)	30 (26.1%)	27 (7.0%)	16 (13.9%)	22 (5.7%)	2 (1.7%)	2 (0.5%)	5 (4.3%)	11 (2.8%)
	**χ**^**2**^ **= 49.3, *p* < .001**		χ^2^ = 0.01, *p* = .924		**χ**^**2**^ **= 32.3, *p* < .001**		**χ**^**2**^ **= 8.63, *p* = .003**		χ^2^ = 1.68, *p* = .194		χ^2^ = 0.66, *p* = .417	
Tests	96 (34.8%)	138 (54.3%)	52 (18.8%)	35 (13.8%)	45 (16.3%)	37 (14.6%)	33 (12.0%)	23 (9.1%)	12 (4.3%)	14 (5.5%)	53 (19.2%)	25 (9.8%)
	**χ**^**2**^ **= 20.5, *p* < .001**		χ^2^ = 2.47, *p* = .116		χ^2^ = 0.31, *p* = .581		χ^2^ = 1.18, *p* = .278		χ^2^ = 0.38, *p* = .535		**χ**^**2**^ **= 9.23, *p* = .002**	
Other	72 (29.3%)	58 (32.8%)	46 (18.7%)	19 (10.7%)	32 (13.0%)	20 (11.3%)	48 (19.5%)	30 (16.9%)	9 (3.7%)	14 (7.9%)	53 (21.5%)	46 (26.0%)
	χ^2^ = 0.59, *p* = .442		**χ**^**2**^ **= 5.02, *p* = .025**		χ^2^ = 0.28, *p* = .598		χ^2^ = 0.45, *p* = .503		χ^2^ = 3.62, *p* = .057		χ^2^ = 1.13, *p* = .287	

a Percentages are not displayed and chi-square test was not performed when cell sizes were < 5. Pan = Pandemic. Pre-pandemic is defined as March 27, 2019 to March 26, 2020 and pandemic is defined as March 27, 2000 to March 26, 2021.

**Table 3.** Referrals after cancer screening inquiry by time and cancer site among general public Cancer Information Service (CIS) users (%), March 2019 – March 2021^a^

	Breast (%)		Cervical (%)		Colorectal (%)		General (%)		Lung (%)		Other (%)	
	Pre-Pan	Pan	Pre-Pan	Pan	Pre-Pan	Pan	Pre-Pan	Pan	Pre-Pan	Pan	Pre-Pan	Pan
N	319	639	113	79	118	80	150	177	24	32	129	105
CDC NBCCEDP	65 (83.3%)	328 (93.4%)	9 (11.5%)	26 (7.4%)	5 (6.4%)	5 (1.4%)	-	-	-	-	-	-
	***χ***^**2**^ **= 8.49, *p* = .004**		*χ*^2^ = 1.45, *p* = .228		***χ***^**2**^ **= 6.97, *p* = .008**		-		-		-	
Health professionals	169 (31.4%)	274 (49.2%)	95 (17.6%)	49 (8.8%)	89 (16.5%)	51 (9.2%)	100 (18.6%)	104 (18.7%)	17 (3.2%)	24 (4.3%)	93 (17.3%)	76 (13.6%)
	***χ***^**2**^ **= 36.19, *p* < .001**		***χ***^**2**^ **= 18.71, *p* < .001**		***χ***^**2**^ **= 13.3, *p* < .001**		*χ*^2^ = .003, *p* = .960		*χ*^2^ = 1.01, *p* = .314		*χ*^2^ = 2.74, *p* = .098	
Organization	176 (55.7%)	514 (79.9%)	35 (11.1%)	34 (5.3%)	55 (17.4%)	41 (6.4%)	34 (10.8%)	46 (7.2%)	7 (2.2%)	8 (1.2%)	26 (8.2%)	28 (4.4%)
	***χ***^**2**^ **= 61.69, *p* < .001**		***χ***^**2**^ **= 10.63, *p* = .001**		***χ***^**2**^ **= 28.61, *p* < .001**		*χ*^2^ = 3.6, *p* = .058		*χ*^2^ = 1.3, *p* = .255		***χ***^**2**^ **= 5.98, *p* = .014**	
Other	124 (42.6%)	170										
(54.3%)	37 (12.7%)	20 (6.4%)	41 (14.1%)	32 (10.2%)	53 (18.2%)	62 (19.8%)	9 (3.1%)	12 (3.8%)	40 (13.7%)	34 (10.9%)		
	***χ***^**2**^ **= 8.27, *p* = .004**		***χ***^**2**^ **= 7.06, *p* = .008**		*χ*^2^ = 2.12, *p* = .145		*χ*^2^ = 0.25, *p* = .618		*χ*^2^ = 0.25, *p* = .619		*χ*^2^ = 1.17, *p* = .280	
No referral	19 (21.8%)	13 (16.5%)	18 (20.7%)	6 (7.6%)	8 (9.2%)	10 (12.7%)	23 (26.4%)	38 (48.1%)	-	-	18 (20.7%)	12 (15.2%)
	*χ*^2^ = .77, *p* = .380		***χ***^**2**^ **= 5.74, *p* = .017**		*χ*^2^ = .51, *p* = .474		***χ***^**2**^ **= 8.36, *p* = .004**		-		*χ*^2^ = 0.85, *p* = .358	

a Percentages are not displayed and chi-square test was not performed when cell sizes were < 5. Pan = Pandemic. Pre-pandemic is defined as March 27, 2019 to March 26, 2020 and pandemic is defined as March 27, 2000 to March 26, 2021.

## Data Availability

These data are collected and managed by NCI. NCI post-doctoral fellows are able to write a project proposal to apply for access to the data.

## Summary Box:

It is well known that the COVID-19 pandemic resulted in the reduction of nonemergent clinical appointments, which included cancer screening tests. However, less is known about how cancer screening information-seeking among the general public may have changed after the onset of the pandemic. Given that cancer screening information-seeking is associated with improved adherence and timeliness of screening, this study aimed to contextualize changes in cancer screening information-seeking following the COVID-19 pandemic in order to optimize communication of cancer information during future crises.
